# Effect of boundary chain folding on thermal conductivity of lamellar amorphous polyethylene†

**DOI:** 10.1039/c9ra07563a

**Published:** 2019-10-18

**Authors:** Yulou Ouyang, Zhongwei Zhang, Qing Xi, Pengfei Jiang, Weijun Ren, Nianbei Li, Jun Zhou, Jie Chen

**Affiliations:** Center for Phononics and Thermal Energy Science, China–EU Joint Lab for Nanophononics, School of Physics Science and Engineering, Tongji University Shanghai 200092 People's Republic of China jie@tongji.edu.cn; Institute of Systems Science and Department of Physics, College of Information Science and Engineering, Huaqiao University Xiamen 361021 People's Republic of China

## Abstract

Thermal transport properties of amorphous polymers depend significantly on the chain morphology, and boundary chain folding is a common phenomenon in bulk or lamellar polymer materials. In this work, by using molecular dynamics simulations, we study thermal conductivity of lamellar amorphous polyethylene (LAPE) with varying chain length (*L*_0_). For a short *L*_0_ without boundary chain folding, thermal conductivity of LAPE is homogeneous along the chain length direction. In contrast, boundary chain folding takes place for large *L*_0_, and the local thermal conductivity at the boundary is notably lower than that of the central region, indicating inhomogeneous thermal transport in LAPE. By analysing the chain morphology, we reveal that the boundary chain folding causes the reduction of both the orientation order parameter along the heat flow direction and the radius of gyration, leading to the reduced local thermal conductivity at the boundary. Further vibrational spectrum analysis reveals that the boundary chain folding shifts the vibrational spectrum to the lower frequency, and suppresses the transmission coefficient for both C–C vibration and C–H vibration. Our study suggests that the boundary chain folding is an important factor for polymers to achieve desirable thermal conductivity for plastic heat exchangers and electronic packaging applications.

## Abstract 2

Boundary chain folding leads to inhomogeneous thermal transport in lamellar amorphous polyethylene with uniform mass density.

## Graphical abstract

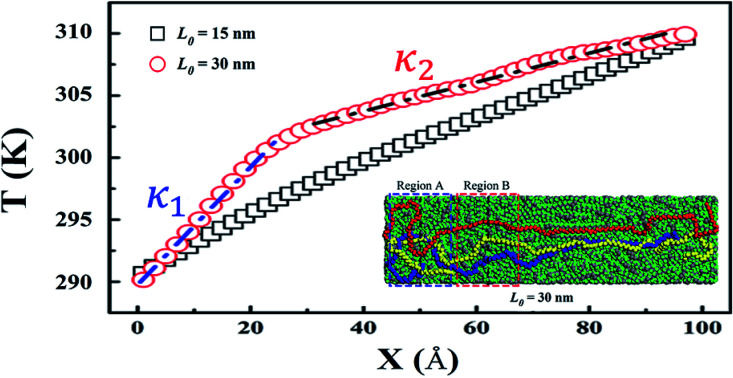

## 1 Introduction

Recent studies suggest that polymers have great potential applications in many fields, such as thermoelectrics,^1,2^ solar cells,^3,4^ light-emitting diodes,^5^ biomedical devices,^6^ nonlinear optical devices,^7,8^ and electronic packaging.^9^ As the most commonly used material for electronic packaging, the heat transfer capability of a polymer film directly determines the heat dissipation and operating performance of the electronic device. In this regard, a comprehensive understanding of the thermal transport properties of amorphous polymers is crucial. The morphology of the surface polymer chain in a bulk polymer is mainly the lamellar chain-folded type, which is formed during the crystal growth process from quiescent melt or solutions of flexible long-chain polymers.^10,11^ Actually, many polymeric materials contain surface chain-folded lamellar and intervening amorphous regions.^12,13^ Due to a lower free-energy barrier compared to the inter-molecular secondary nucleation, the chain folding is attributed to intra-molecular secondary crystal nucleation at the crystal growth front.^10^ During the growth of polymer, the configuration of adjacent chain-folding can limit the thickness of polymer.^14^

The bulk amorphous polymers typically have a low thermal conductivity around 0.1–1.0 W m^−1^ K^−1^ at room temperature,^15,16^ which is two to three orders of magnitude lower than that of crystals.^17,18^ The random orientation of the polymer chains and the weak inter-chain couplings are the two main mechanisms responsible for the low thermal conductivity.^19^ Proper alignment of polymer chains is found to be an effective method to enhance the polymer's thermal conductivity, which can achieve more than two orders of magnitude enhancement for amorphous polyethylene (PE).^17,20^ Using molecular dynamics simulations, Henry *et al.*^21,22^ found that thermal conductivity of a single PE chain can reach up to 300 W m^−1^ K^−1^, which is almost as high as that of some inorganic semiconductors and metals.

The morphology of polymer chains also plays an important role on the thermal conductivity of polymer. Zhang *et al.*^23^ used PE as a model system to systematically investigate the fundamental relationship between the molecular morphology and thermal conductivity in bulk amorphous polymers. They found that thermal transport through covalent bonds dominates the effective thermal conductivity in polymers, and the thermal conductivity of rigid rod-like polymer can be increased by more than one order of magnitude, compared to that of polymers with very soft backbone. For lamellar amorphous polyethylene (LAPE), Ma *et al.*^24^ found that stronger confinement and less entanglement lead to a smaller thermal conductivity. However, the impact of boundary chain-folding on thermal transport in lamellar polymer has not been understood yet. This is particularly relevant for lamellar polymer because the surface-to-volume-ratio is rather large^7^ due to the limited film thickness, and the influence of chain folding cannot be neglected.

In this work, we study the thermal conductivity of LAPE by using molecular dynamic simulation to elucidate the effect of chain folding on thermal transport. Our results show that the chain folding results in the non-uniform local temperature gradient in LAPE. To describe the thermal transport property of the whole system in the presence of non-uniform temperature gradient, the validity of using effective thermal conductivity is discussed. The physical origin for the non-uniform local thermal conductivity is revealed by comparing various factors in different regions, including chain morphology, orientation, and vibrational spectrum. Our study provides a solid understanding for the influence of chain configuration on the thermal transport in LAPE, which will be beneficial for the design of polymer based materials for thermal management applications.

## 2 Model and simulation

To take into account the variation of polymer chain length in experiments, we first construct PE chains with varying lengths, which are sufficiently extended and quasi-straight by randomly changing the torsion angles (see Fig. S1(a) in ESI†). Here the chain length refers to the total length of the polymer chain when it is fully extended (straight). The chain length distribution satisfies the Gaussian distribution 
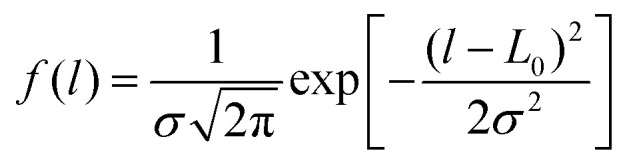
 where *L*_0_ is the average length, and *σ* is the standard deviation. Table 1 summarizes the detailed configurations for various PE chains with different lengths with a given standard deviation of *σ* = 1 nm. As the total number of atoms in our study is fixed (about 27 000), the number of chains will decrease with the increase of *L*_0_. We then use the PACKMOL software^25,26^ to randomly place the PE chains into a sufficiently large box (50 nm × 10 nm × 10 nm), which allows for the construction of the initial lamellar structure (see Fig. S1(b) in ESI†).

**Table 1:** Detailed configurations for polyethylene chains with varying average length and constant standard deviation (*σ* = 1 nm) in molecular dynamics simulation. The initial size of the simulation domain is fixed as 50 nm × 10 nm × 10 nm for all cases

Average chain length (*L*_0_)	5 nm	10 nm	15 nm	20 nm	25 nm	30 nm
Number of chains (*N*)	225	112	75	56	45	37
Total number of atoms	27 000	26 880	27 000	26 940	27 000	26 640
Final density after NPT relaxation (g cm^−3^)	0.69	0.71	0.70	0.68	0.68	0.72
Simulation box size after NPT relaxation: *X* (nm)/*Y* (nm)/*Z* (nm)	19.8/4.0/4.0	20.1/3.9/4.0	20.0/4.0/4.0	20.9/3.9/3.9	19.5/4.1/4.1	19.6/4.0/4.0

After preparing the initial structure, molecular dynamics simulations are performed to study the dynamics of the system by using the large-scale atomic/molecular massively parallel simulator (LAMMPS) software.^27^ The adaptive inter-molecular reactive empirical bond order (AIREBO) potential^28^ is used to describe inter- and intra-molecular interactions between carbon and hydrogen atoms, which has been widely used to study the polymer systems in literature.^21,22^ The time step is 0.2 fs, and the force cutoff distance is set as 10 Å. Periodic boundary conditions are adopted in all directions.

**Fig. 1:**
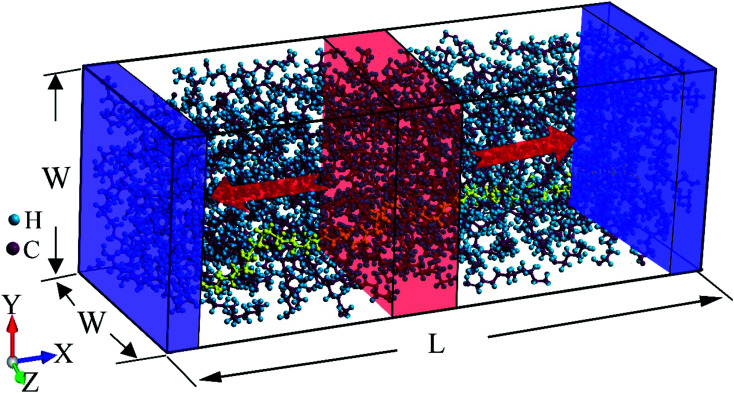
Non-equilibrium molecular dynamics simulation setup for the lamellar amorphous polyethylene (LAPE) chains. The size of the simulation box is around *L* = 20 nm and *W* = 4 nm. An individual polyethylene chain is highlighted in yellow. The high temperature thermostat (one red region with thickness of 10 Å) is applied to the center of the simulation domain, while the low temperature thermostat (two blue regions with thickness of 5 Å) is applied to the two ends. Periodic boundary conditions are used in all directions. This setup has a mirror symmetry that results in the bidirectional heat flow.

The initial structure is relaxed by performing equilibrium molecular dynamics (EMD) simulation with the isothermal-isobaric (NPT) ensemble at 1 atm and 300 K for 2 ns, after which the thermal equilibrium and structure relaxation have been achieved (see Fig. S2 in ESI†). More details about the structure generation are included in the ESI.† EMD simulations with canonical (NVT) ensemble at 300 K are then performed for another 1 ns. A typical relaxed atomic structure of LAPE is shown in Fig. 1.

After structure relaxation, we then carry out the non-equilibrium molecular dynamics (NEMD) simulations to compute the thermal conductivity of LAPE. As shown in Fig. 1, two Nosé-Hoover thermostats at 310 K and 290 K are applied to the center and two ends of the simulation domain, respectively, in order to establish the temperature gradient along the LAPE. The lattice thermal conductivity *κ* of the amorphous PE is then calculated as:1
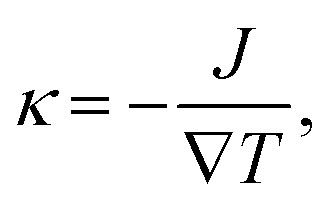
where ∇*T* is the temperature gradient along the length (*X*) direction, and *J* is the heat flux defined as the energy transported across unit area per unit time^29^2
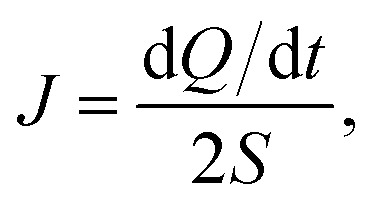
where d*Q*/d*t* is the average rate of the energy injection and extraction in the thermostated regions, and *S* is the cross-sectional area. The factor of two in the denominator accounts for the bidirectional heat flow (Fig. 1) due to the use of periodic boundary condition. We record the temperature profile along the *X*-direction by dividing the simulation domain into slices with a uniform width of 2 Å. The temperature gradient is then calculated by fitting the linear portion of the temperature profile. The NEMD simulations are performed long enough (2 ns) to ensure the non-equilibrium steady state in which the temperature profile and heat flux are time-independent. The final thermal conductivity results are averaged over three independent NEMD simulations with different velocity distribution as initial conditions. It should be noted that the thermal transport behaviors in nanomaterials are significantly different from that of bulk materials. For instance, in one-dimensional nanotubes and nanowires,^30–33^ and two-dimensional graphene,^34^ the peculiar size dependent thermal conductivity has been reported. Therefore, we focus on the effect of boundary chain folding on the thermal conductivity of PE for the finite-size system, which allows us to discover some new phenomena different from bulk PE.

## 3 Result and discussion

### 3.1 Effective thermal conductivity

To test the AIREBO potential for thermal conductivity of PE, we calculate the thermal conductivity of bulk PE, which is found to be 0.34 W m^−1^ K^−1^ at room temperature. This result falls in the range of 0.22–0.37 W m^−1^ K^−1^ reported by the literature study^35–37^ for the bulk PE. Furthermore, the final structures for different LAPE samples with varying lengths have a similar density about 0.70 g cm^−3^ after NPT relaxation (Table 1), which is in good agreement with the reported density 0.68–0.73 g cm^−3^ at ambient condition.^38^

**Fig. 2:**
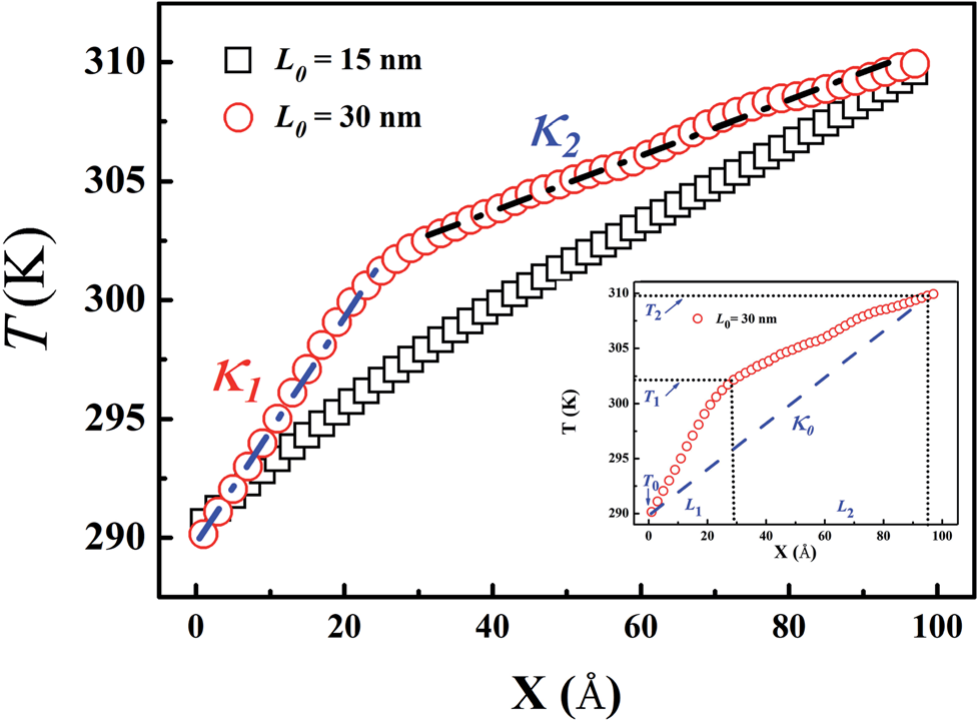
Typical temperature profile for LAPE samples with different average length (*L*_0_) and constant standard deviation (*σ* = 1 nm). The results are averaged over two mirror symmetry regions. The square and circle represent the temperature profile for *L*_0_ = 15 nm and *L*_0_ = 30 nm, respectively. In the presence of non-uniform temperature gradient for *L*_0_ = 30 nm, *κ*_1_ and *κ*_2_ denote the local thermal conductivity in the boundary and internal region, respectively. The inset depicts the calculation of effective thermal conductivity *κ*_0_ for *L*_0_ = 30 nm based on the artificial uniform temperature gradient between two ends.

Fig. 2 shows the typical temperature profile along the length direction for LAPE with different average length (*L*_0_) and constant standard deviation (*σ* = 1 nm). For short PE chains (*L*_0_ = 15 nm), a linear temperature profile (square in Fig. 2) is established in the whole region between the heat source and heat sink. In contrast, the temperature profile for long PE chains (*L*_0_ = 30 nm) is no longer linear across the whole region, but becomes piecewise linear, exhibiting a two-segment kinked characteristic (circle in Fig. 2). Besides, the temperature gradient in the boundary region (*X* < 20 Å) is greater than that in the internal region (*X* > 20 Å). Furthermore, we repeat the simulations for *L*_0_ = 30 nm with Langevin heat bath, and the same non-uniform characteristics in temperature profile are also observed (see Fig. S4 in ESI†).

According to the Gaussian distribution, the length of various chains mainly falls into the range from *L*_0_ − 3*σ* to *L*_0_ + 3*σ*. When the upper bound of the chain length (*L*_0_ + 3*σ*), for instance *L*_0_ = 15 nm, is smaller than the length of the simulation domain (*L* = 20 nm), each PE chain can be placed into the simulation box so that no boundary chain folding occurs. On the other hand, when the length of PE chains is longer than the simulation domain, they have to be folded at the boundary in order to fit into the simulation box. The two-segment kinked temperature profile obviously suggests that the boundary chain folding has a remarkable impact on the thermal transport in LAPE.

In order to quantify the effect of boundary chain folding, we calculate the thermal conductivity of LAPE with varying average length *L*_0_. When a uniform linear temperature gradient (square in Fig. 2) is established, thermal conductivity can be directly calculated according to eqn (1) for the whole region. In the presence of boundary chain folding, however, the two-segment piecewise linear temperature gradient (circle in Fig. 2) occurs, and thus only local thermal conductivity in each linear segment can be calculated by eqn (1) instead. As shown in Fig. 2, *κ*_1_ and *κ*_2_ represents the local thermal conductivity for the boundary and internal region, respectively.

Here, a natural question to be asked is how to define the *effective* thermal conductivity for the whole system in the presence of non-uniform temperature gradient? This problem has been seldomly emphasized in literatures as most materials are homogeneous along the temperature gradient direction. For instance, one widely used experimental technique to measure thermal conductivity is the thermal bridge method,^34,39,40^ which directly relates thermal conductivity of sample to the temperature change in the heater and sensor assuming a linear temperature gradient across the sample. Since the non-uniform temperature gradient is not considered in the experimental model, the second question is the validity of such calculation (*κ*_0_ in the inset of Fig. 2) and underlying assumption.

To address these questions, let us start with the relationship between the thermal resistance (*R*) and thermal conductivity (*κ*) *κ* = *L*/(*SR*), where *L* and *S* denotes the length and cross sectional area for the system of interest, respectively. Then, one can define the local thermal resistance as:3
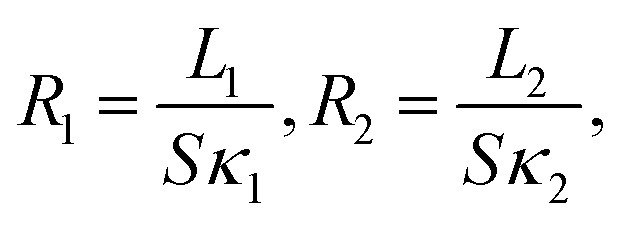
where *L*_1_ and *L*_2_ denotes the length of boundary and internal segment with linear temperature gradient, respectively. Similar to the electric circuit case, one has to rely on the serial or parallel connection model in order to get the total resistance. As the boundary segment and internal segment are placed adjacently along the temperature difference direction, it is reasonable to assume serial heat conduction channels rather than parallel connection. Therefore, the effective (total) thermal resistance *R*_eff_ is given by the serial connection rule as *R*_eff_ = *R*_1_ + *R*_2_. Correspondingly, the effective thermal conductivity *κ*_eff_ for the whole system can be expressed as:4
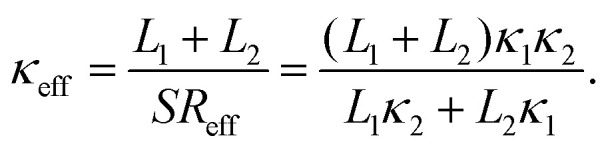


Based on eqn (4), one immediate derivation is that *κ*_eff_ ≈ *κ*_2_ in the limit when *L*_1_ ≪ *L*_2_. In other words, when the length of the boundary segment is much smaller than that of the internal segment, the assumption of a uniform linear temperature gradient is well justified as the temperature gradient for the internal segment is equivalent to the temperature gradient across the whole system in this limit case. This condition can be satisfied in most experimental measurements when the contact (boundary) region is much smaller than the internal suspended region.

Interestingly, we further prove rigorously in ESI† that *κ*_eff_ given by eqn (4) is actually identical to *κ*_0_ in which one fictitious temperature gradient is used in the calculation (inset in Fig. 2), regardless of the emergence of the non-uniform temperature gradient. That is to say, even in the presence of non-uniform temperature gradient among different segments, if one assumes serial connection between each heat dissipation segment, it is essentially equivalent to define an *effective* uniform temperature gradient established across the whole system. Similarly, for thermal conductance, we can also define an *effective* thermal conductance analogue to the effective thermal conductivity (see ESI† for more detail).

**Fig. 3:**
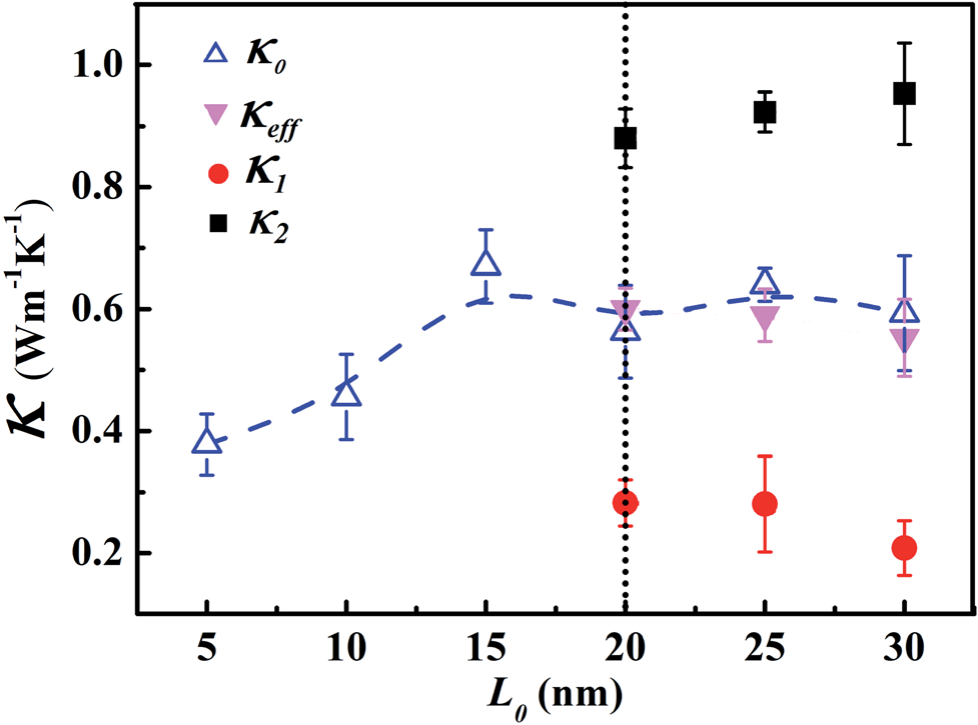
Room temperature thermal conductivity of LAPE as a function of the average chain length (*L*_0_) with fixed *σ* = 1 nm. *κ*_0_ (empty triangle), *κ*_1_ (solid circle), and *κ*_2_(solid square) are computed by eqn (1), while *κ*_eff_ (solid triangle) is computed by eqn (4). The dashed lines are drawn to guide the eye.

To verify such equivalence numerically, we compute two types of effective thermal conductivity *κ*_eff_ (eqn (4) with two linear fittings) and *κ*_0_ (eqn (1) with one linear fitting) for different LAPE samples with varying length (Fig. 3). For short length samples (*L*_0_ < 20 nm), only *κ*_0_ can be defined as there is only one uniform temperature gradient. Besides, thermal conductivity exhibits an increasing trend with the average chain length, which is consistent with the previous theoretical study on amorphous polymer at short length scale.^41^ For longer samples (*L*_0_ ≥ 20 nm) where boundary chain folding takes place, *κ*_eff_ and *κ*_0_ overlap with each other within the statistical error, and both of them show no obvious length dependence. Moreover, the local thermal conductivity for the internal region (*κ*_2_) is consistently higher than that for the boundary region (*κ*_1_) for the same *L*_0_. Obviously, the emergence of non-uniform local thermal conductivity hinders the continuous growth of effective thermal conductivity with length in LAPE. These results suggest that the boundary chain folding has a remarkable impact on thermal conductivity of LAPE.

### 3.2 Effect of chain length distribution

**Fig. 4:**
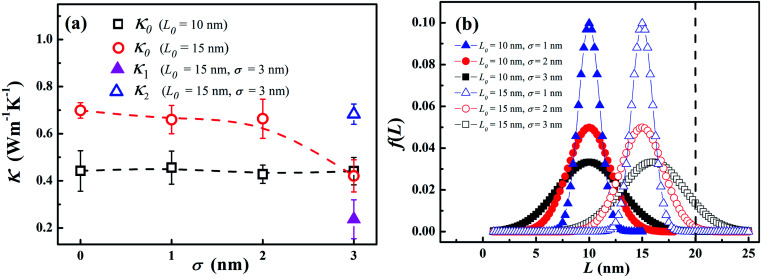
(a) Room temperature thermal conductivity of LAPE *versus* the standard deviation (*σ*) of the chain length distribution. In the sample with *L*_0_ = 15 nm and *σ* = 3 nm, local thermal conductivities (*κ*_1_, *κ*_2_) appear due to the folding of the polyethylene chains. (b) The chain length distribution in the polyethylene samples for *L*_0_ = 10 nm (solid symbols) and *L*_0_ = 15 nm (empty symbols), respectively. The dashed lines are drawn to guide the eye.

In order to explore the effect of chain length distribution on the thermal transport in LAPE, we further compute thermal conductivity *κ*_0_ of PE chains with varying *σ* for two typical length *L*_0_. As shown in Fig. 4(a), when *L*_0_ is equal to 10 nm, *κ*_0_ is almost constant (∼0.4 W m^−1^ K^−1^) when varying *σ* from 0 to 3 nm. For *L*_0_ = 15 nm, *κ*_0_ is higher (∼0.7 W m^−1^ K^−1^) and remains constant when increasing *σ* up to 2 nm, but drops to a lower value when *σ* = 3 nm, in which the boundary chain folding process takes place. By computed the local thermal conductivity for *L*_0_ = 15 nm and *σ* = 3 nm, we find the thermal conductivity for the internal region *κ*_2_ (empty triangle in Fig. 4(a)) is still close to that before the emergence of chain folding, but thermal conductivity for the boundary region *κ*_1_ (solid triangle in Fig. 4(a)) is much lower (only ∼0.2 W m^−1^ K^−1^), leading to the reduced effective thermal conductivity according to eqn (4). Therefore, we can conclude that the variation of *σ* value has no impact on thermal conductivity of LAPE unless the boundary chain folding occurs.

The variation of thermal conductivity with *σ* can be understood by analyzing the distribution function of PE chain length. As shown in Fig. 4(b), the upper bound for chain distribution for the sample with *L*_0_ = 10 nm (solid symbols) does not exceed the length of simulation domain (*L* = 20 nm) for all *σ* values, which means that each chain has enough space to extend. As we fix the average length *L*_0_, with the variation of *σ* value, the chain length of some PE chains decreases while that of others increases, so that the thermal conductivity for the whole system is size independent. This is also the case for the sample with *L*_0_ = 10 nm and *σ* < 3 nm. However, for the sample with *L*_0_ = 15 nm and *σ* = 3 nm, there is a notable portion of PE chains whose chain length is longer than the length of simulation domain. Consequently, these long PE chains have to be folded at the boundary in order to fit into the simulation box, which gives rise to the non-uniform local thermal conductivity.

### 3.3 The influence of morphology

**Fig. 5:**
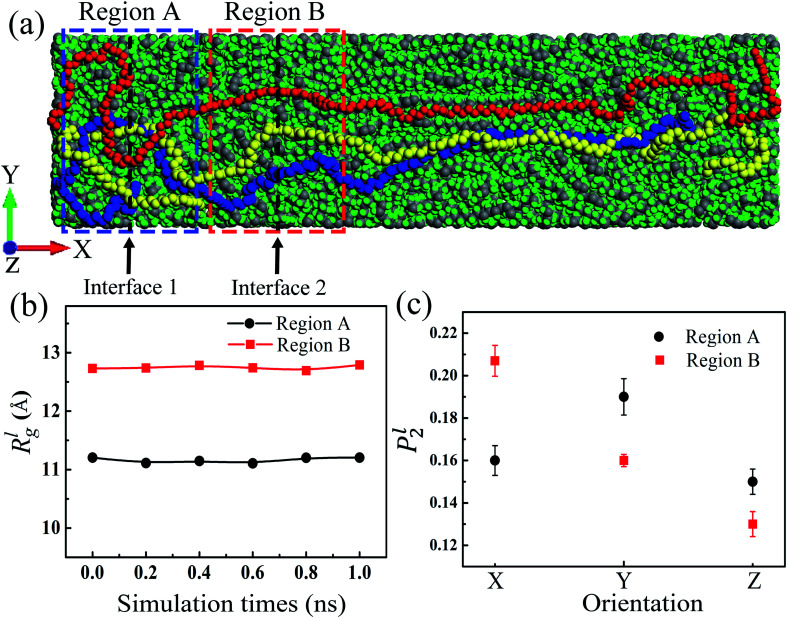
(a) A schematic diagram for the LAPE sample with *L*_0_ = 30 nm and *σ* = 1 nm. Three polyethylene chains (only carbon backbone) are highlighted in red, yellow and blue, respectively. Region A and B denote the boundary and internal region, respectively. (b) The local radius of gyration *R*_*g*_^*l*^*versus* simulation time in region A (circle) and region B (square). (c) The local orientation order parameter *P*_2_^*l*^ in different regions.

As the mass density is uniform along the length direction of LAPE, the emergence of non-uniform local thermal conductivity suggests that the boundary chain folding induced morphology change could be an important factor. To directly visualize the chain morphology in these samples, we show in Fig. 5(a) a typical snapshot of the molecular conformation for LAPE with a long length (*L*_0_ = 30 nm and *σ* = 1 nm). Here three individual chains are highlighted with different colors. It is clearly shown that each PE chain is more extended in the internal region (region B), while it becomes more twisted in the boundary region (region A).

In order to quantify the morphology change, we compute the radius of gyration (*R*_*g*_) of PE chains, which is one of the most important parameters to characterize the conformation of amorphous PE. The radius of gyration for an individual PE chain is defined as:^24,42^5
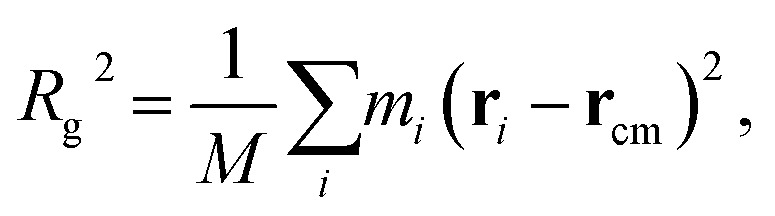
6
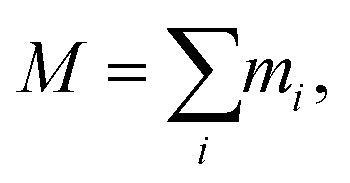
7
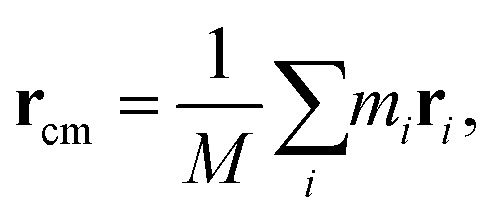
where *M* is the total mass, **r**_cm_ is the center of mass position, and *m*_*i*_ and **r**_*i*_ are the mass and position for atom *i*, respectively. For a single polymer chain, the summations in eqn (5)–(7) include all atoms on the same chain. For a polymer chain with the fixed length, a larger *R*_*g*_ value means more extended chain morphology. As the chain folding phenomenon mostly takes place in the boundary region, in order to capture the morphology change in a specific region, we further compute the *local* radius of gyration *R*_*g*_^*l*^, which is obtained by restricting the summations in eqn (5)–(7) only to the atoms on the same chain inside a specific region and then averaging over all polymer chains in the same region.

Since the morphology of a PE chain is mainly determined by carbon atoms on the backbone,^43,44^ we only include the carbon backbone in order to simplify the calculation of *R*_*g*_^*l*^. In order to record the structure variation, we run EMD simulation for another 1 ns after the structure relaxation process. Fig. 5(b) shows the variation of *R*_*g*_^*l*^*versus* time for the sample with *L*_0_ = 30 nm and *σ* = 1 nm in different regions. For each region, *R*_*g*_^*l*^ is almost time-independent, confirming that the polymer structure has been fully relaxed. Besides, *R*_*g*_^*l*^ in region A is consistently lower than that in region B. This difference means PE chains near the boundary are more entangled as a result of the chain folding, which is consistent with the result that the local thermal conductivity for the boundary region is lower than that for the internal region (Fig. 3). Such relation suggests that the entangled PE chains suppress the thermal transport in LAPE. Previous studies also have shown that more extended chain morphology with larger radius of gyration is favorable for more efficient thermal transport in amorphous polymers.^45^

Although the radius of gyration can measure the degree of entanglement for polymer chains, it contains no information about the chain alignment direction, which is an important factor that can affect the polymer's thermal conductivity.^46^ To this end, we introduce here the orientational order parameter *P*_2_ that can be used to characterize the segment alignment along different directions defined as:^47–49^

8 *P*_2_ = 1.5 < (**e**_*i*_⋅**e**_*x*_)^2^ > −0.5,

where **e**_*x*_ is the unit vector in the direction of the *X*-axis, *Y*-axis, or *Z*-axis, the angular bracket means the ensemble average, and **e**_*i*_ is the direction vector for a line segment connecting two nearest neighbors on the same chain given by9
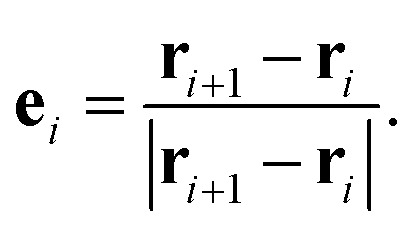


A value of *P*_2_ = −0.5 means that the orientation is perpendicular to the selected direction, and *P*_2_ = 1 means that the orientation is parallel to the selected direction. Similar to the calculation of *R*_*g*_^*l*^, we compute the *local* orientational order parameter *P*_2_^*l*^, including only those atoms in a specific region.

As shown in Fig. 5(c), *P*_2_^*l*^ in region B exhibits the largest value along the *X*-axis (temperature bias direction), but *P*_2_^*l*^ in region A has a peak value along the *Y*-axis. In particular, along the *X*-axis, *P*_2_^*l*^ in region B is larger than that in region A. These results indicate that PE chains in the internal region are mostly aligned along the length (temperature bias) direction, while the alignment of PE chains in the boundary region deviates substantially from the thermal transport direction due to the chain folding process. As the heat conduction in polymer is mainly through the covalently bonded backbone,^37^ the conformable alignment of polymer chains along the temperature bias direction will greatly promote the thermal transport in polymer systems. This explains the origin for the non-uniform local thermal conductivity in the presence of chain folding, and the lower thermal conductivity in the boundary region. Our results are consistent with the previous study which reported the improvement on the chain alignment, achieved by the mechanical stretching, can increase the polymer's thermal conductivity.^46^ Therefore, in addition to the morphology change, the chain alignment is another factor responsible for the higher thermal conductivity in the internal region.

### 3.4 Vibrational spectrum analysis

In dielectric solids, thermal energy is mainly carried by the atomic vibrations. In crystalline materials, the atomic vibration can be described by the phonons. The reduced phonon lifetime is one of the mechanisms responsible for the reduction of thermal conductivity in crystalline solids,^50,51^ due to the enhanced phonon scatterings. However, for amorphous materials, such as the amorphous polyethylene in our work, the phonon properties (such as the group velocity and lifetime) are no longer well-defined due to the lack of lattice periodicity.^52^ Vibrational spectral analysis is an important tool to understand the thermal transport mechanism in various nanostructures.^53–55^ However, few studies have analyzed the influence of the chain morphology on vibrational properties of polymer. To probe the underlying mechanisms for the effect of chain folding on thermal conductivity, we compute the vibrational density of states (VDOS) as^56–58^10
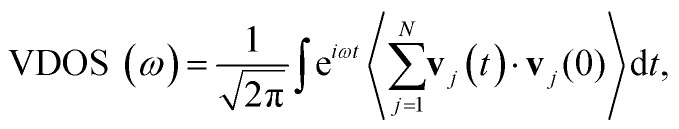
where **v**_*j*_(*t*) is the time-dependent velocity for atom *j*, and *N* is the total number of atoms in a specific region.

**Fig. 6:**
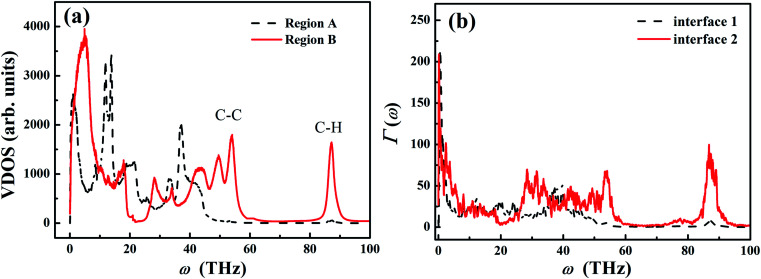
(a) Vibrational density of states (VDOS) of polyethylene chain in different regions. (b) Spectral transmission coefficient *Γ*(*ω*) at *T* = 300 K in different regions computed from the NEMD simulations.

Fig. 6(a) shows the VDOS in different regions (as shown in Fig. 5(a)) for the sample with *L*_0_ = 30 nm and *σ* = 1 nm. For the extended PE chains in region B, VDOS exhibits the high frequency peak around 90 THz, which is a characteristic for CH_2_ stretching vibration.^59^ Moreover, the VDOS peak at ∼50 THz is the characteristic G-peak of carbon material,^60^ and the low-frequency peaks (<30 THz) originates from the flexibility (angular bending and dihedral rotations) of the carbon–carbon bonds.^56^ Compared to the carbon–carbon bond, carbon–hydrogen bonds are associated with higher vibrational frequencies due to the lighter weight of hydrogen atoms.^56^

As a result of the chain folding in region A, the high frequency spectrum (∼50 THz) shifts to the lower frequency, and the ultrahigh frequency vibrations (∼90 THz) are significantly suppressed in the PE chains. Such frequency shift is qualitatively consistent with the previous molecular dynamics study by Brayton *et al.*,^61^ in which they found that the vibration spectrum shifted to the lower frequency when the structure of PE changed from crystal to semi-crystal to amorphous structure. In our study, the PE chains in region A are less extended and more confined compared to those chains in region B. The chain folding restricts the vibration of the covalent C–C bond and reduces the order of chain alignment.

In order to further understand the effect of chain folding on thermal transport properties of LAPE, we calculate the frequency spectrum of transmission coefficient *Γ*(*ω*) defined as^62,63^11
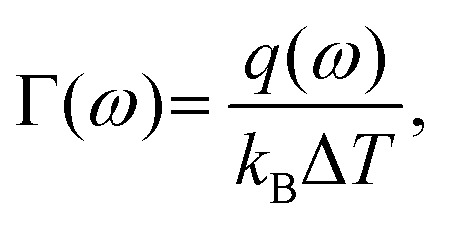
where *k*_B_ is the Boltzmann constant, Δ*T* is the temperature difference, and *q*(*ω*) is the spectral heat flux across the imaginary interface (Fig. 5(a)). The heat flux can be computed from NEMD simulations according to^62,64^12
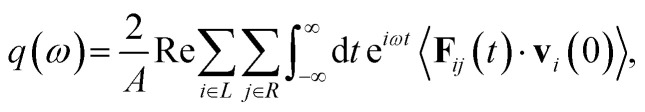
where *A* is interface cross sectional area, **v**_*i*_ is the velocity of atom *i*, **F**_*ij*_ is the force between the atom *i* and *j*, and the symbol *L* and *R* denotes the left and right side of the imaginary interface, respectively.

Fig. 6(b) shows the calculation results of *Γ*(*ω*) from NEMD simulation. Compared to the transmission spectrum for region B (solid line), the original characteristic peaks ∼30 THz and 50 THz have been greatly suppressed in region A (dashed line). In particular, the transmission of C–H vibration at very high frequency (∼90 THz) is also forbidden in region A. Therefore, the boundary chain folding induces substantial suppression of the transmission for both C–C vibration and C–H vibration, which leads to the reduced local thermal conductivity in the boundary region.

## 4 Conclusion

In summary, we have studied the thermal transport behavior of the lamellar amorphous polyethylene with varying chain lengths by using molecular dynamics simulations. For short chain length, a uniform temperature gradient can be established and thermal conductivity increases with the average chain length. With the increase of average chain length, the phenomenon of chain folding starts to occur at the boundary, leading to the non-uniform temperature gradient (thus local thermal conductivity) along the heat transport direction. The local thermal conductivity for the boundary region is found to be notably lower than that for the internal region, and the effective thermal conductivity of the whole system shows no obvious length dependence. Compared to the boundary chain folding region, we find that, based on the analysis of the chain morphology, the polyethylene chains in the internal regions are more extended and better aligned along the heat transport direction. Furthermore, the vibrational spectrum analysis reveals that the boundary chain folding shifts the vibrational spectrum to lower frequency, and suppresses the transmission coefficient for both C–C vibration and C–H vibration, leading to the reduced local thermal conductivity in the boundary region. Our study provides a microscopic insight to the understanding of fundamental thermal transport behavior in lamellar amorphous polymers.

## Conflicts of interest

There are no conflicts of interest to declare.

## Supplementary Material

RA-009-C9RA07563A-s001
