# Supplementary material for: Effect of boundary chain folding on thermal conductivity of lamellar amorphous polyethylene
Source: RSC Adv. 2019 Oct 18;9(57):33549–57. doi: 10.1039/c9ra07563a (PMC9073277; doi:10.1039/c9ra07563a)
Supplement: RA-009-C9RA07563A-s001 [file RA-009-C9RA07563A-s001.pdf]

Electronic Supplementary Material (ESI) for RSC Advances.
This journal is © The Royal Society of Chemistry 2019

Supporting Information

Effect of Boundary Chain Folding on Thermal Conductivity of Lamellar Amorphous Polyethylene

Yulou Ouyang[1], Zhongwei Zhang[1], Qing Xi[1], Pengfei Jiang[1], Weijun Ren[1], Nianbei Li[2], Jun Zhou[1], and Jie Chen[1,*]

[1]Center for Phononics and Thermal Energy Science, China–EU Joint Lab for Nanophononics, School of Physics Science and Engineering, Tongji University, Shanghai 200092, People's Republic of China

[2]Institute of Systems Science and Department of Physics, College of Information Science and Engineering, Huaqiao University, Xiamen 361021, People’s Republic of China

*Corresponding author: Email: jie@tongji.edu.cn

1. Construct amorphous polyethylene for NEMD simulation

To construct amorphous polyethylene for NEMD simulation, we use the following procedure (Fig. S1): (1) Generate polyethylene chains with different lengths. (2) Use PACKMOL software to randomly load polyethylene chains into an initial simulation box with fixed size. The chain length distribution satisfies Gaussian distribution. (3) After minimization, an NPT run at 300 K is used to generate amorphous polyethylene with fully relaxed structure.

Based on LAMMPS software, we use “iso” keywords during the relaxation process to couple all 3 diagonal components together when pressure is computed (hydrostatic pressure), and dilate/contract the dimensions together. Thus the ratio of length to width of the equilibrated structure is consistent with that of the initial simulation box. Therefore, in order to ensure that all polyethylene chains can be put into the box, we set the size of the initial simulation box as 50 nm × 10 nm × 10 nm. After relaxation, in each samples, the size of the final equilibrated structure reaches a size about 20 nm × 4 nm × 4 nm. When the chain length of polyethylene is longer than the length of final box size (~ 20 nm), there is not enough space for polyethylene to extend, thus these long chains will be folded. Therefore, in this way, the chain folding phenomenon can be realized by changing the chain length of the polyethylene in the initial structure.

To ensure that the morphologies of polyethylene obtained from such a procedure is well equilibrated, we monitored the energy, temperature and the simulations box size for each system during the NPT ensemble. Here, take the samples (L_0 = 30 nm,

σ= 1 nm) as an example (see Fig. S2). The plateaus are reached after the relaxation process.

2. Effective and Fourier's law thermal conductivity

In order to explore the difference between the thermal conductivity calculated according to the series rule of thermal resistance and the Fourier's law when there is non-linear thermal conductivity in the sample, we decomposed the Eq.(4), $\kappa_{eff} = \frac{L_1 + L_2}{SR_{eff}} = \frac{(L_1 + L_2)\kappa_1\kappa_2}{L_1\kappa_2 + L_2\kappa_1}$, in the main text. Here, we take the temperature distribution of the sample (L_0 = 30 nm and σ = 1 nm) as example (inset in Fig. 2). If we assume that the temperature distribution is linear (blue dash line in the illustration of Fig.2), κ_0 can be expressed as $\kappa_0 = -\frac{J}{\frac{T_2 - T_0}{L_1 + L_2}}$. According the Eq.(1) in the main text, κ_{eff} can be decomposed to:

$$\kappa_{eff} = \frac{(L_1 + L_2)\kappa_1\kappa_2}{L_1\kappa_2 + L_2\kappa_1} = \frac{(L_1 + L_2) \times \left(-\frac{J}{\frac{T_1 - T_0}{L_1}}\right) \times \left(-\frac{J}{\frac{T_2 - T_1}{L_2}}\right)}{L_1 \times \left(-\frac{J}{\frac{T_2 - T_1}{L_2}}\right) + L_2 \times \left(-\frac{J}{\frac{T_1 - T_0}{L_1}}\right)} = -\frac{\frac{L_1L_2J^2(L_1 + L_2)}{(T_1 - T_0)(T_2 - T_1)}}{\frac{L_1L_2J(T_2 - T_0)}{(T_1 - T_0)(T_2 - T_1)}}$$

$$= -\frac{J}{\frac{T_2 - T_0}{L_1 + L_2}} = \kappa_0 \tag{S1}$$

where $(T_2 - T_0)/(L_1 + L_2)$ is the temperature gradient.

Similarly, the thermal conductance G can be written as:

$$G = \frac{dQ/dt}{\Delta T} \tag{S2}$$

where dQ/dt and ΔT is the average rate of the energy injection and extraction in the thermostated regions and the temperature differecnce, respectively. Similar to κ_0 (inset in Fig. 2), G_0 can be expressed as $G_0 = \frac{dQ/dt}{T_2 - T_0}$. According to the relationship between the thermal conductance and thermal resistance ($G = \frac{1}{R}$), the effective thermal conductance G_{eff} for the whole system can be expressed as $G_{eff} = \frac{1}{R_{eff}} = \frac{1}{R_1 + R_2}$. Therefore, G_{eff} can be decomposed to:

$$\begin{aligned} G_{eff} &= \frac{1}{R_1 + R_2} = \frac{1}{\frac{1}{G_1} + \frac{1}{G_2}} = \frac{1}{\frac{1}{\frac{dQ/dt}{T_1 - T_0}} + \frac{1}{\frac{dQ/dt}{T_2 - T_1}}} \\ &= \frac{1}{\frac{(T_1 - T_0) + (T_2 - T_1)}{dQ/dt}} \\ &= \frac{dQ/dt}{T_2 - T_0} = G_0 \end{aligned} \tag{S3}$$

Based on the above derivations, we can conclude that two kinds of thermal conductance (G_{eff} and G_0) and thermal conductivity (κ_{eff} and κ_0) are equivalent, if serial heat conuction channels between different segments are assumed.

3. Temperature profile of the LAPE

Figure S3 shows the temperature profiles for samples with different average chain length (L_0) with fixed standard deviation (σ = 1 nm). An obvious kinked temperature gradient is observed for samples with L_0 = 20 nm and 25 nm. In addition, we further

verify the non-uniform temperature profile by applying different heat bath in molecular dynamics simulations. Figure S4 shows the temperature profile of the long sample (L_0 = 30 nm, σ = 1 nm) with Langevin thermostat, in which the two-segment tempeature profile is also observed. This result confirms that the non-uniform temperature gradient phenomenon is caused by the structure of polymer chain itself, but not by the specific thermostat used in the simulations.

Figure

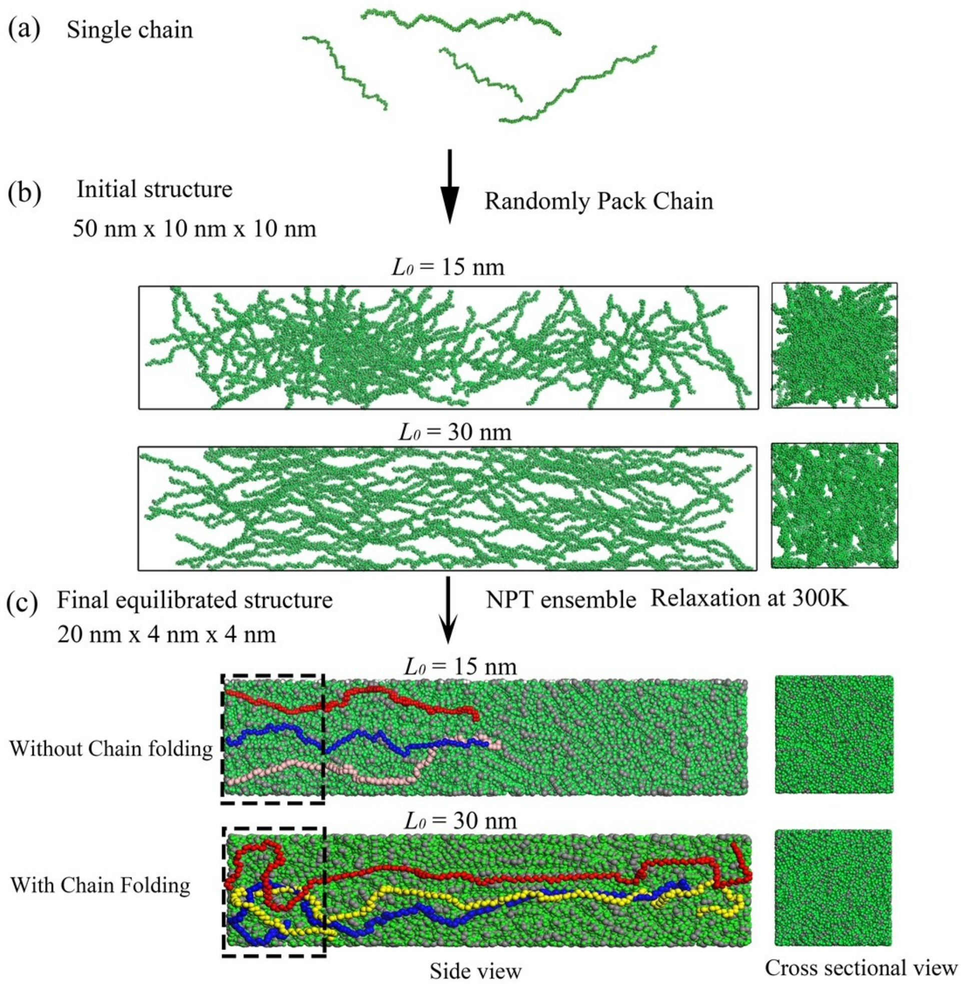

Figure S1. Schematic graph for the generation of lamellar amorphous polyethylene

(LAPE) structure. (a) Illustration of single molecular chain of PE. (b) Schematic diagram of two typical initial structures (L_0 = 15 nm, σ = 1 nm and L_0 = 30 nm, σ = 1 nm) in our simulations. Many PE chains that chain length satisfies Gaussian distribution with average length L_0 and standard deviation σ are packed into the initial simulation box with L = 50 nm and W = 10 nm. (c) The stabilized structures are equilibrated using a NPT ensemble at 300K and 1 atm. The size of all the equilibrated structures is around L = 20 nm and W = 4 nm. An individual polyethylene chain is highlighted in yellow.

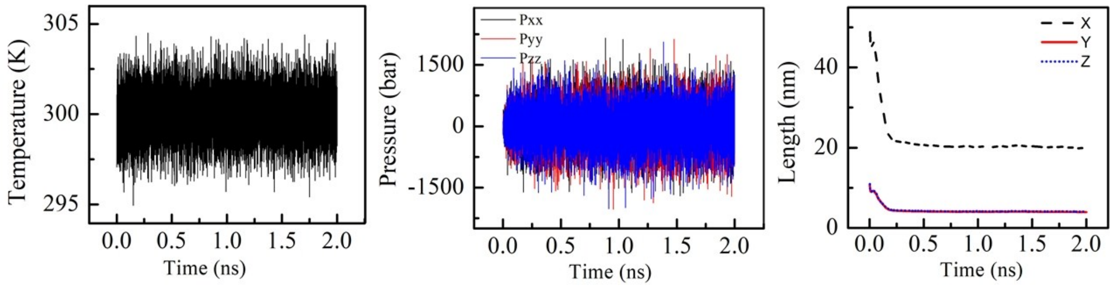

Figure S2. The temperature, stress/pressure and the simulation box size of the whole system for the polyethylene samples (L_0 = 30 nm and σ = 1 nm) during the relaxation process.

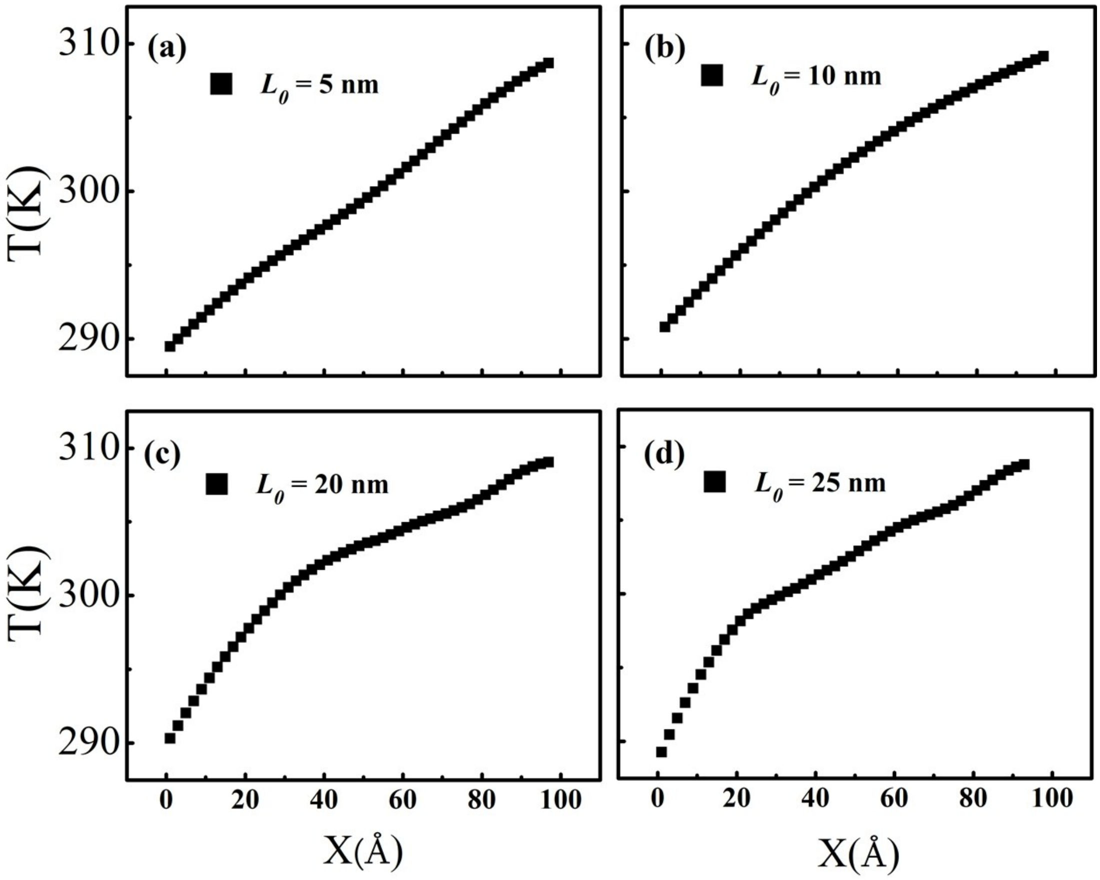

Figure S3. Temperature profile for polyethylene chains with varying average length (L_0) and constant standard deviation (σ = 1 nm).

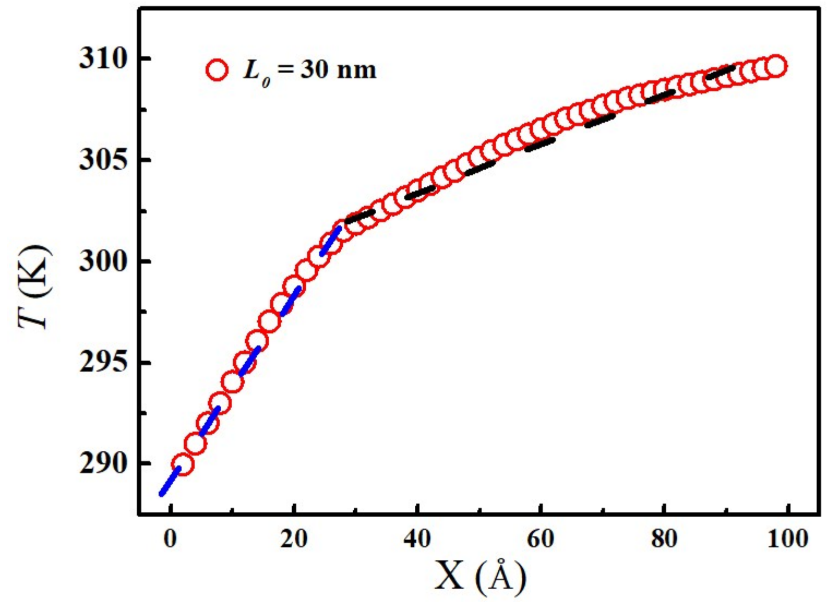

Figure S4. The temperature profile of the long sample (L_0 = 30 nm, σ = 1 nm) by applying Langevin thermostat.
